# THE RANCHO BERNARDO STUDY (RBS) OF HEALTHY AGING: A RICH RESOURCE FOR STUDYING AGING IN WOMEN

**DOI:** 10.1093/geroni/igz038.1289

**Published:** 2019-11-08

**Authors:** Linda McEvoy, Linda K McEvoy, Gail A Laughlin, Donna Kritz-Silverstein, Richelle Bettencourt, Jaclyn Bergstrom

**Affiliations:** 1 University of California San Diego, San Diego, California, United States; 2 Department of Radiology, Department of Family Medicine and Public Health, University of California, San Diego, La Jolla, California, United States; 3 Department of Family Medicine and Public Health, University of California, San Diego, La Jolla, California, United States

## Abstract

RBS is an ideal cohort to study healthy aging in women and to examine sex differences in aging. Initiated in 1972, RBS enrolled 82% of adult residents of Rancho Bernardo, a suburb of San Diego. Residents were white, and middle to upper-middle class. Participants have been followed via 12 research clinic visits at ~4 year intervals and 32 mailed surveys. RBS contains detailed assessment of cardiometabolic disease risk factors, bone health, biomarkers, physical ability, cognitive function, health and reproductive history, medications, behaviors (smoking, drinking, diet, exercise) and psychosocial measures. Of the 6726 participants, 54% are women and 65% were aged ≥50 at enrollment. Vital status is known for 91% of the cohort; overall mortality is 71%. Death certificates have been coded for cause of death for 91% of decedents. We will discuss contributors to healthy longevity in RBS women (average age of death 86.4 yrs; 29% lived to age ≥90).

